# Interpretable Conditional Recurrent Neural Network for Weight Change Prediction: Algorithm Development and Validation Study

**DOI:** 10.2196/22183

**Published:** 2021-03-29

**Authors:** Ho Heon Kim, Youngin Kim, Yu Rang Park

**Affiliations:** 1 Department of Biomedical Systems Informatics College of Medicine Yonsei University Seoul Republic of Korea; 2 Noom Inc New York, NY United States

**Keywords:** explainable AI, interpretable AI, mHealth, obesity, behavior modification, artificial intelligence, development, validation, weight, intervention

## Abstract

**Background:**

In recent years, mobile-based interventions have received more attention as an alternative to on-site obesity management. Despite increased mobile interventions for obesity, there are lost opportunities to achieve better outcomes due to the lack of a predictive model using current existing longitudinal and cross-sectional health data. Noom (Noom Inc) is a mobile app that provides various lifestyle-related logs including food logging, exercise logging, and weight logging.

**Objective:**

The aim of this study was to develop a weight change predictive model using an interpretable artificial intelligence algorithm for mobile-based interventions and to explore contributing factors to weight loss.

**Methods:**

Lifelog mobile app (Noom) user data of individuals who used the weight loss program for 16 weeks in the United States were used to develop an interpretable recurrent neural network algorithm for weight prediction that considers both time-variant and time-fixed variables. From a total of 93,696 users in the coaching program, we excluded users who did not take part in the 16-week weight loss program or who were not overweight or obese or had not entered weight or meal records for the entire 16-week program. This interpretable model was trained and validated with 5-fold cross-validation (training set: 70%; testing: 30%) using the lifelog data. Mean absolute percentage error between actual weight loss and predicted weight was used to measure model performance. To better understand the behavior factors contributing to weight loss or gain, we calculated contribution coefficients in test sets.

**Results:**

A total of 17,867 users’ data were included in the analysis. The overall mean absolute percentage error of the model was 3.50%, and the error of the model declined from 3.78% to 3.45% by the end of the program. The time-level attention weighting was shown to be equally distributed at 0.0625 each week, but this gradually decreased (from 0.0626 to 0.0624) as it approached 16 weeks. Factors such as usage pattern, weight input frequency, meal input adherence, exercise, and sharp decreases in weight trajectories had negative contribution coefficients of –0.021, –0.032, –0.015, and –0.066, respectively. For time-fixed variables, being male had a contribution coefficient of –0.091.

**Conclusions:**

An interpretable algorithm, with both time-variant and time-fixed data, was used to precisely predict weight loss while preserving model transparency. This week-to-week prediction model is expected to improve weight loss and provide a global explanation of contributing factors, leading to better outcomes.

## Introduction

In the last 30 years, the prevalence of global obesity has increased [1]. The increasing prevalence has been observed in all countries, whether low-and-middle or high-income [2]. In the United States, the prevalence has increased in children as well as adults [3]. Because large populations with obesity are at risk of comorbid conditions, such as cardiovascular disease, gastrointestinal disorders, type 2 diabetes, and psychological issues that may increase the risk of mortality and affect their daily lives [4], this public health problem poses burdens from both an economic and health perspective [5].

As an alternative to conventional obesity management, which requires visiting medical institutions and medical staff, more attention has been given to smartphone-based interventions due to their accessibility (providing low-cost service to large populations anytime and anywhere) [6,7]. With the increase in obesity management using smartphones, a number of apps aimed toward obesity are being developed [8]. Many existing apps have collected patient-generated health data over time, including passive or active data. These data may help predict outcomes because they are from a heterogeneous population and can capture high-resolution information outside clinical settings [9-11]; however, the use of these data for weight loss prediction has not been sufficiently researched using an obesity management app.

To forecast medical outcomes such as weight loss, artificial intelligence (AI) has been adopted in the medical field [12-14]. Specifically, in AI modeling, recurrent neural networks (RNNs) have been used with longitudinal data [12,15,16]. Recently, to make RNNs transparent, more attention has been focused on explainable AI for critical safety applications in the medical field [17]. However, although time-fixed data such as demographic data, depend on each patient, and it is important in the medical field, no attempt has been made to use both serial and time-fixed data while preserving model transparency [16]. Within the same context that doctors apply while taking patient histories and monitoring consecutive laboratory examinations, it is necessary to use both types of medical data. From 2 types of data, explainable AI can act as an interpreter for the results or findings and translate them into a format that can easily be understood while maintaining performance for timely feedback in obesity management [18,19]. Furthermore, the explanation from this model is a prerequisite for new insight into patterns in obesity management.

In this study, we aimed to (1) develop a predictive model by utilizing both time-variant and time-fixed data for weight loss prediction in a mobile-based intervention using interpretable AI and (2) explore the factors affecting weight loss or gain based on app usage.

## Methods

### Mobile App

Noom (Noom Inc) is a mobile app that provides various lifestyle-related logs and is available for Android and Apple devices. The app provides interventions based on (1) food logging, (2) exercise logging, (3) weight logging, (4) in-app group activities, (5) article reading, and (6) communication with coaches through messaging [20]. Once users sign up and install the app, they are required to record their initial status information such as weight, BMI, and target weight. The coach encourages users to record their food intake, daily exercise, and weekly weight [20]. Furthermore, passive data, such as the number of steps taken, are automatically collected and saved in order to track users’ behaviors.

### Study Design and Participants

We obtained anonymous and deidentified log data of Noom users in the United States from August 8, 2013 to August 8, 2019 for a retrospective study. This study adhered to the TRIPOD [21] statement on reporting predictive models.

From a total of 93,696 users in the coaching program, we excluded users who did not take part in the 16-week weight loss program. From this group, we ruled out users who had not entered weight or meal records for the entire 16-week program. Although the coach encouraged users to enter their meal and weight log daily, those with incomplete records were considered users who did not successfully receive the intervention; moreover, we could not evaluate their outcomes in the model for weight loss at 16 weeks. We included users who had a target weight that was below the initial weight to exclude users with the purpose of weight gain. In addition, we excluded those whose height did not fall between 125 cm and 230 cm (n=19,129).

**Figure 1:**
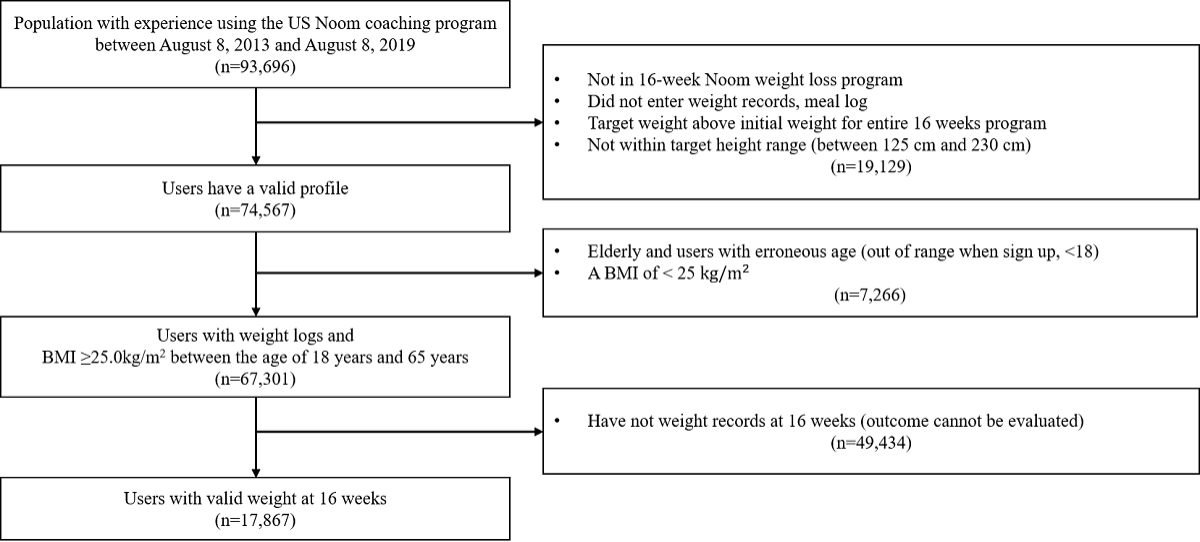
Selection flow for eligible users.

In users with a valid profile, we included only adult users (range: 18 to 65 years old) and excluded older adults over 65 years old and users under 18 years old. Furthermore, only overweight and obese users (BMI ≥25 kg/m^2^ based on the definitions provided by the Center for Disease Control and Prevention [22]) were included in our prediction model to target users with a need for weight loss (n=67,301). Finally, we identified 17,867 eligible users with 16 weeks of weight records for analysis to evaluate the outcome, presented as predicted weight (Figure 1).

### Development of a Weight Loss Prediction Model

We developed an interpretable RNN model by modifying the reverse time attention model (RETAIN) [16}. As the purpose of the RETAIN model is to predict the risk of cardiac disease using sequential data on categorical data such as diagnosis (International Statistical Classification of Diseases, Tenth Revision), we improved the model to (1) give leverage to continuous variables such as weight input frequency and time-fixed variables such as user profile and (2) convert this into a regression model for the prediction of weight after 16 weeks while preserving the linearity of the model for interpretability.

**Figure 2:**
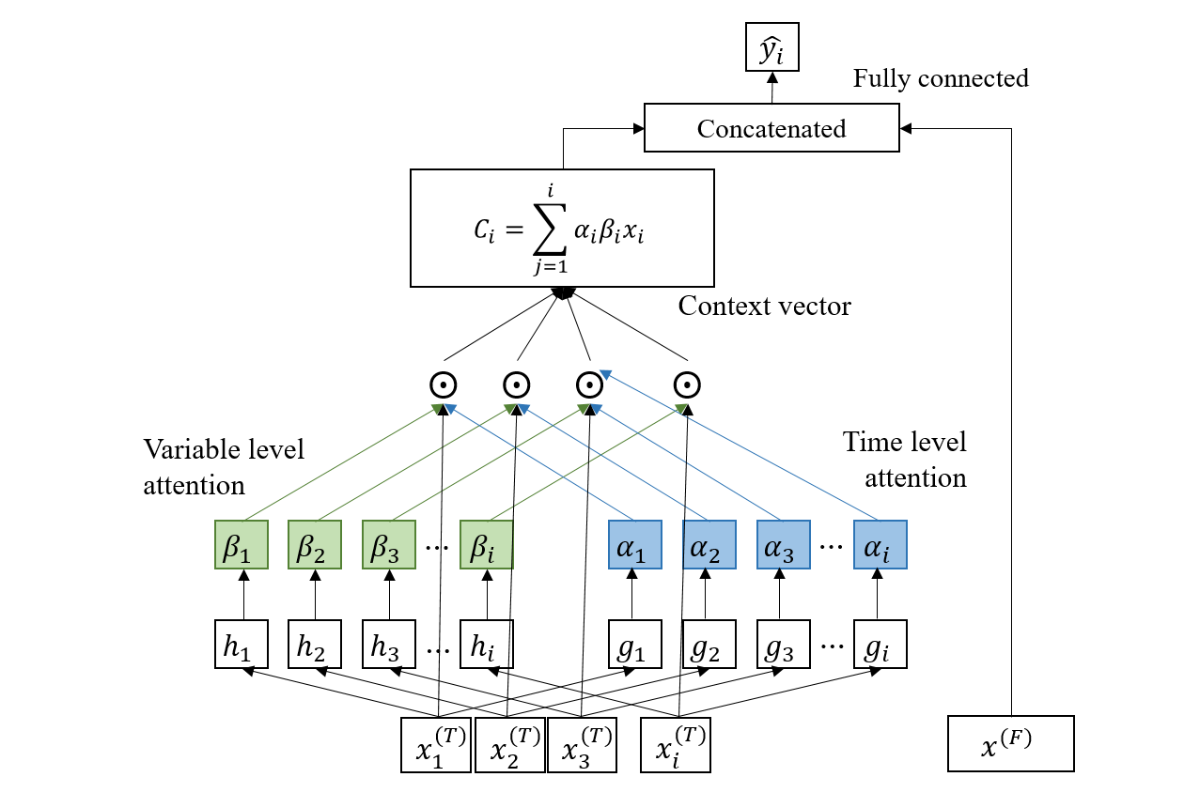
Model architecture.

We modified the prediction model to (1) consider time-fixed variables by concatenating initial status as auxiliary inputs, (2) use continuous variables by removing the embedding layer, and (3) convert this into a regression model by not using the activation function for classification (Figure 2).

Assuming we have *N* timeseries related to app usage with *i* length, where 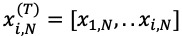 and 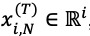 *k* time-fixed variables where 
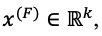
 and a target series *y* of length *i*, where **y**=[*y*_1_,…,*y*_i_], and 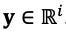. By stacking *N* timeseries, we define a multivariate input series 
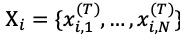
 and contemporary multivariate input vector is denoted at time *i* by 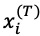. Given *X*_i_ and *x*^(F)^, we leverage 2 types of data for our model to predict contemporary target values using deep learning 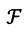, namely 
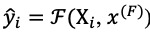
. The concatenation operation is denoted by ⊕ and element-wise multiplication is denoted by ⊙. *A*_:i_ is denoted by slice 1-dimensional tensor *A* from first index to *i*th index (Multimedia Appendix 1).

We attempted to predict the participant’s weight after a 16-week intervention using the output vector *y*_i_ ∈ {0, ∞} by concatenating *c_i_* from time-variant data and *x*^(F)^ and applying a linear transformation with *w* weight vector, where *w* ∈ 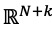. This is expressed as follows:


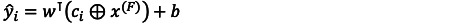


where *c_i_* denotes the context vector *c_i_* ∈ 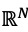 and is the sum of the time-level variables 
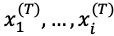
 weighted by attention α_i_, β_i_. Time-level attention weighting α_i_ is a scalar value that reflects the relative importance of the data at time *i*, and it ranges from 0 to 1. β_i_ is a vector with *N* length that explains the importance of each value of 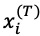 within *i* time.

Consistent with RETAIN, interpretation of the weight prediction model involves getting the time-level attention weighting and variable-level attention weighting from each RNN *g*_i_, *h*_i_ [16]. Therefore, the equation can be rewritten, and the contributions of the predicted value of the model can be calculated as follows:


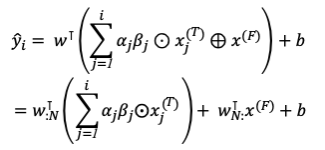


Therefore, the contribution of the *N*th time-variant variable ω(*ŷ*_i_, *x_jN_*)^(^*^T^*^)^ and that of the *m*th time-fixed variable ω(*ŷ*_i_, *x_m_*)^(^*^F)^* can also be written as


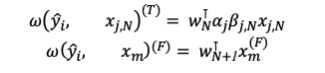


where each contribution coefficient of the time-variant variable is 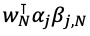, and each contribution coefficient of the time-fixed variable is 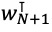.

We subsequently generated 3 dimensions of time-variant variables based on app usage patterns (window, week, variables) to provide immediate feedback if users performed behaviors that infringed on weight loss from week to week, and to improve model performance, and for real-time prediction of future target value, we fixed all elements of *y* as the weight at 16 weeks.

Furthermore, we leveraged information on the shape of the weight loss trend week to week. We used shape-based timeseries clustering on the weight logs; individuals’ weights were assigned into clusters at each time for real-time prediction. The weight loss trajectory can be labeled at each time point and can represent the historical shape of weight loss, not actual weight (kilograms) from the first week to each subsequent week. This trajectory does not require the outcome of the model. Therefore, the real-time model can leverage a user’s historical shape of weight loss at each time. To exclude the fact that weight (the outcome of the model) is directly used as a feature, we normalized each weight log by converting their means to 0, and the variance to 1. Then, we used the clustering number in the prediction process. Dynamic time warping was used to measure the distance between user weight loss trajectories; we used *k*-means clustering with this metric. We found 5 to be the optimal number of clusters. For individuals, a weight loss cluster was assigned for each time. An example of weight loss is included in Multimedia Appendix 2.

### Model Evaluation With Cross-Validation Procedure

The performance of the regression model was evaluated using the mean absolute percentage error (MAPE) [23]. MAPE was calculated for the entire intervention (from week 1 to week 16) to identify model accuracy. Furthermore, to evaluate the consistency of the model’s performance, 5-fold cross-validation (training set: 70% and validation set: 30%) was performed.

### Identification of Contribution Coefficients

We identified the contribution coefficients by exploring the time-level attention weighting and variable-level attention weighting for a window of 16 weeks to ascertain the contributions of week 1 to week 16 in predicting weight. Furthermore, we explored the attention weighting of the predictive model by calculating the average of the attention weightings used for predicting weight in test data.

### Ethics

The study was approved by the institutional review board at Advarra (CR00123125). The anonymous and deidentified nature of the retrospective log data made obtaining informed consent unnecessary.

## Results

A total of 17,867 eligible users were included in this study. Their average age was 43.2 years (SD 10.8), and almost all users were female (16,460/17,867, 92.2%). The mean initial BMI was 33.7 kg/m^2^, and users lost a mean of 4.6 kg (SD 4.8). Compared to their initial weight, a majority (5908/17,867 33.1%) of users lost 5% to 10% of their initial weight, followed by users who lost 2% to 5% of their initial weight (4894/17,867, 27.4%) (Table 1).

**Table 1:** Demographic characteristics of eligible users.

Variables		Eligible users (n=17,867)
Age (years), mean (SD)		43.2 (10.8)
**Gender, n (%)**		
	Female	16,470 (92.2)
	Male	1397 (7.8)
Height (cm), mean (SD)		166.4 (7.5)
Initial weight (kg), mean (SD)		93.4 (17.8)
Initial BMI (kg/m^2^), mean (SD)		33.7 (5.8)
Last weight (kg), mean (SD)		88.8 (17.3)
Weight loss^a^ (kg), mean (SD)		4.6 (4.8)
**Outcome, n (%)**		
	Gained >2%	693 (3.9)
	Stable	4075 (22.8)
	Loss of 2%-5%	4894 (27.4)
	Loss of 5%-10%	5908 (33.1)
	Loss of 10%-15%	1983 (11.1)
	Loss >15%	314 (1.8)

^a^Weight loss = initial weight – final weight.

**Figure 3:**
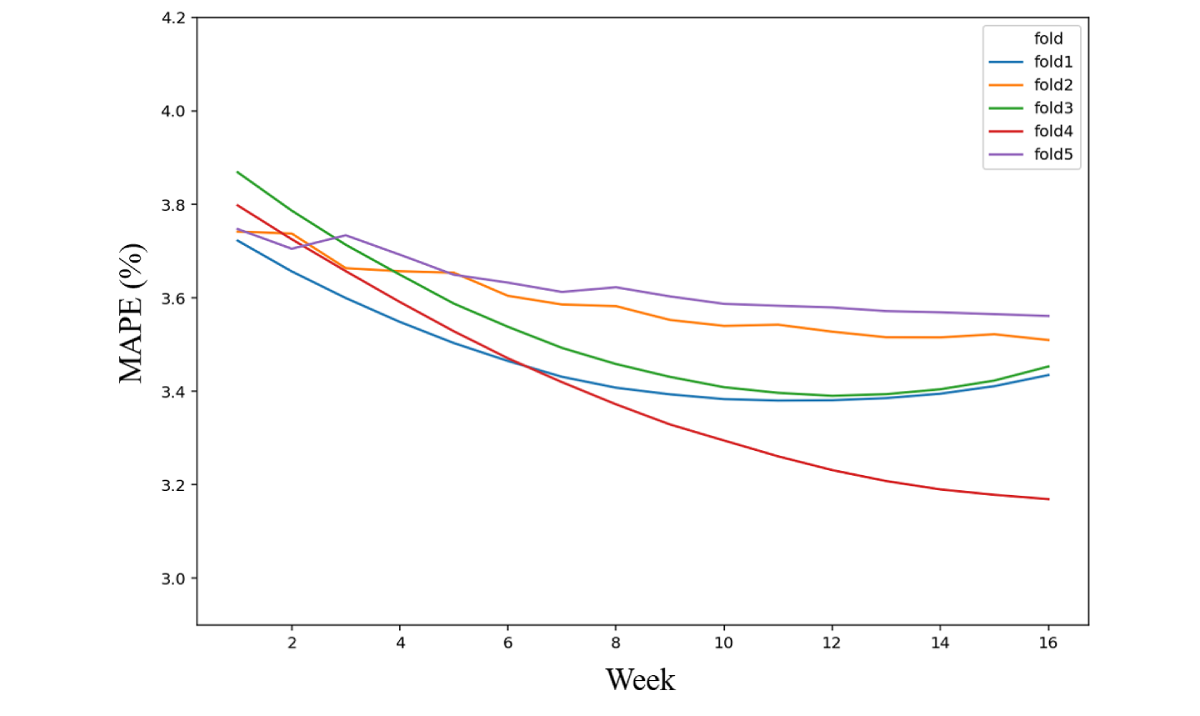
Mean absolute percentage error (MAPE) of model in each 5-fold cross-validation.

The overall MAPE of the model was 3.50%. One, 8, 14, and 16 weeks after the coaching program, the model MAPEs were 3.78%, 3.45%, 3.41%, and 3.45%, respectively. Furthermore, the results of cross-validation showed that the error rates of the predicted value decreased as coaching weeks went by in folds 2, 4, and 5 (Figure 3). However, in folds 1 and 3, the MAPE increased to 3.45%.

**Figure 4:**
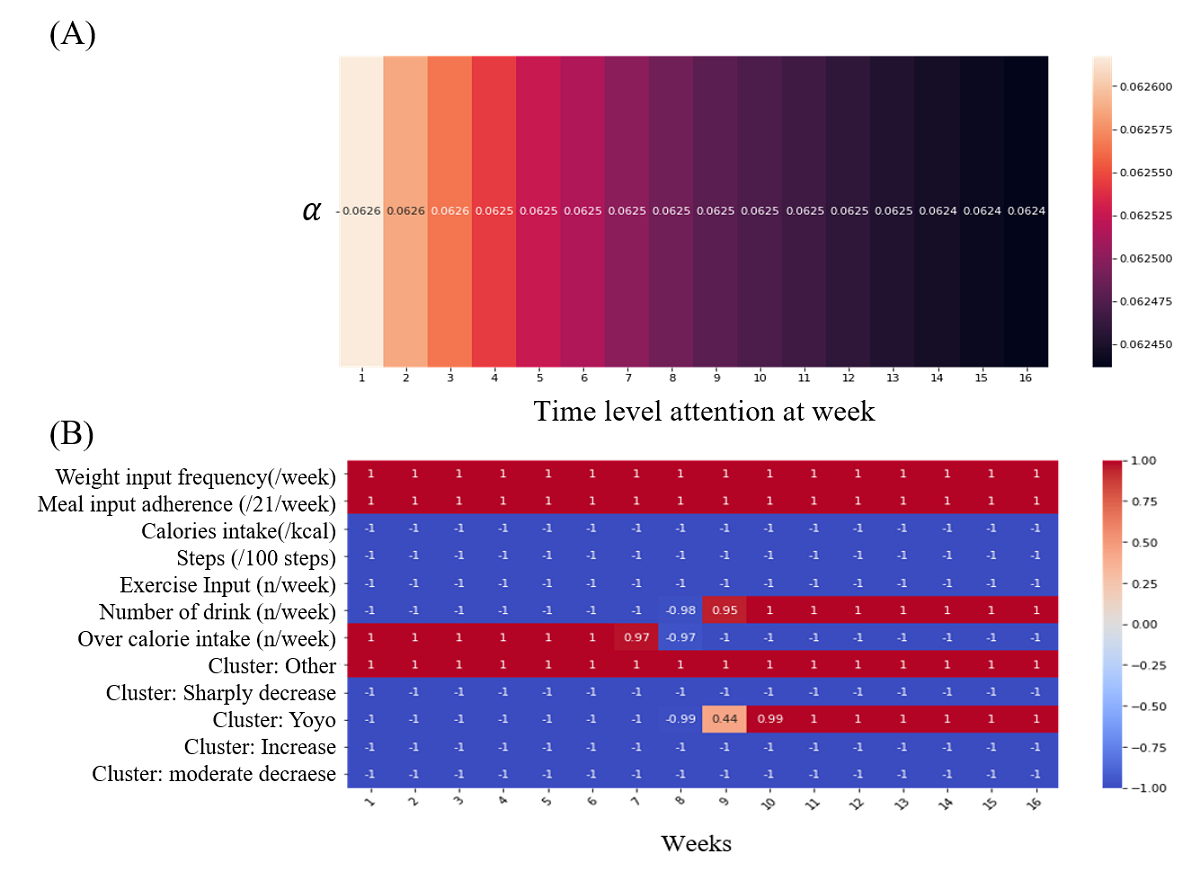
(A) Time-level attention α and (B) variable-level attention β of each variable for the 16-week coaching program.

The overall time-level attention weightings showed that the weight per time was almost evenly distributed as 0.0625; however, the attention weightings gradually decreased over time toward the last week of the coaching program (Figure 4A). The variable level attention weightings of each variable remained at 1 or –1 consistently for 16 weeks; however, the cluster assignment for yo-yo trajectories started off as negative during the first 8 weeks, then became positive in the subsequent 8 weeks (Figure 4B).

The time-variant weightings, including weight input frequency, meal input adherence, and exercise input frequency, had values of –0.340, –0.372, and 0.248, respectively. Among the cluster assignments, a sharp decrease in weight loss trajectory had a weighting of 1.066. Among the time-fixed variables, initial weight highly contributed to predicting weight (0.935). Furthermore, users whose initial weight was in the overweight range were expected to have a lower weight (–0.085). Those whose weights were from obese class I, class II, and class III were expected to have higher weights (0.084, 0.072, and 0.097, respectively) (Table 2).

**Figure 5:**
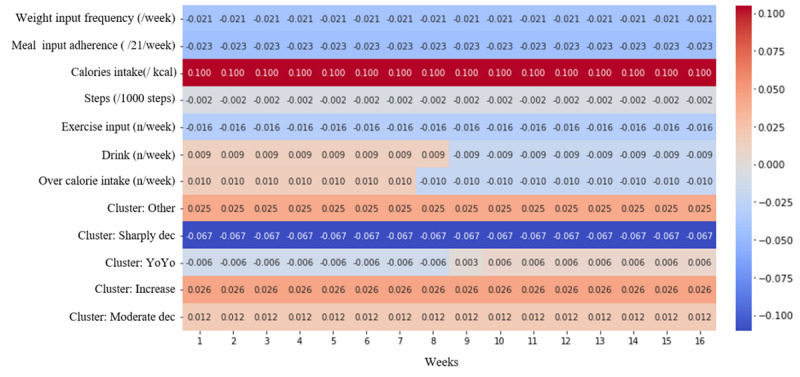
Contribution coefficients for predicting weight change in each week for each variable.

For the contribution coefficient, weight input frequency, meal input adherence, steps, exercise input, and a sharply decreased weight loss trajectory had negative contribution coefficients. Among them, the sharply decreased trajectory had the most negative contribution coefficient (–0.066), followed by the weight input frequency, meal input frequency, and exercise input frequency (–0.021, –0.0232, and –0.0155, respectively). The caloric intake per kilocalorie had a positive contribution coefficient (0.098). Among the trajectories, assignment into the yo-yo group had a negative contribution coefficient (–0.0059) in the first week, however, this became a positive coefficient after 8 weeks (Figure 5).

**Table 2:** Weightings for time-variant and time-fixed variables in matrix w.

Variables			Weightings
**Time-variant**			
	Weight input frequency (n/week)		–0.340
	Meal input adherence (%/week)		–0.372
	Exercise inputs frequency (n/week)		0.248
	Calorie intake (kcal/per days)		–1.597
	Steps (/1000 daily steps)		0.031
	Alcohol drink (n/week)		–0.150
	Over-calorie intake (n/week)		0.164
	**Weight loss trajectories (if assigned)**		
		Sharp decrease	1.066
		Moderate decrease	–0.188
		Yo-yo	0.094
		Increase	–0.422
		Other	0.395
**Time-fixed**			
	**Gender**		
		Female	0.160
		Male	–0.091
	Age (years)		0.002
	Height (cm)		0.013
	Initial weight (kg)		0.935
	**Obesity class (if assigned)**		
		Overweight	–0.085
		Obese class I	0.084
		Obese class II	0.072
		Obese class III	0.097

## Discussion

### Principal Results

In this study, a transparent RNNs model was constructed utilizing both timeseries and time-fixed data for weight loss prediction after a mobile-based intervention targeting obesity. Currently, the explanation methods based on attention mechanisms are referred to as explaining individual predictions because attention can be generated in each person by providing a contributing role in prediction. However, our research first demonstrated the connection between individuals and a global pattern with homogenous length timeseries. Through the identification of time-level and variable-level attention weighting, we identified that weight, meal, and exercise inputs are important factors for weight loss in the context of mobile apps. Furthermore, the findings regarding time-level attention weighting may suggest that constant use of the app is important in mobile-based interventions. In individual profiles, we identified that being male and being overweight were relatively negative contributing factors to weight after the mobile program intervention.

In addition, this predictive model considered both time-variant variables, such as weekly adherence to the app, and time-fixed variables, such as the initial status of users to predict week-to-week weight change. Recently, interpretable RNN architecture has been proposed in machine learning areas [16,24-26]. However, the simple application of this architecture may be difficult in research problems involving classification or regression, such as those in medical fields where demographic information is important for prediction. To address this limitation, we modified the RETAIN model while preserving model transparency using conditional RNNs by adding auxiliary inputs such as basic patient information. Our results, which predicted weight based on interpretation of both demographic status and app usage patterns, showed how each variable was important in predicting weight change each week.

Finally, based on the lifelog data of approximately 17,000 users, we found that a deep learning model that takes advantage of the sequential nature of lifelog data was able to perform well in the general population. Research on models for weight loss prediction has been conducted in fields such as bariatrics or pharmacology on small populations; therefore, it is difficult to generalize the findings of these previous studies [12,27] to the general population. Since we used a general population for this study, these findings may be applied to a general and heterogeneous group.

### Interpretable Algorithm

It is important for end-users such as the patient or clinician to interpret and understand the AI system because erroneous predictions are especially risky and expensive [28]. As black-box machine learning models are frequently employed due to the importance of prediction in critical contexts (such as within the medical field), the demand for transparency in AI, from end-users to research staff, is increasing [29,30]. This has led to a strong interest in explainable AI, which can provide details or reasons to make its functioning clear or easy to understand [17]. Explainable AI research to make RNNs transparent has been conducted [16,24-26]; however, it is difficult to directly adopt these algorithms for problems in medical fields without patient profiles because time-fixed variables have not been properly handled. Therefore, we modified the interpretable AI model to handle both types of data while preserving transparency, and our work can be a cornerstone of medical research in discovering new insights. In addition, contribution coefficients from the model can provide intuitive insight into why the model’s predicted values differed compared to actual data of users’ behaviors and weight change from week to week [16].

### Timely Feedback and Contributing Factors to Weight Loss

We found that weighting was equally distributed each week with a value of 0.0625 (Figure 4); however, this indicated that the initial usage of the app was slightly more important than later usage (from 0.0626 to 0.0624). Given that time-level attention weighting is relative, and the sum of these is 1, the importance of each week is almost equivalent because the weight loss program continued for 16 weeks (0.0625≒1/16). Therefore, this implies that each week in the diet program is nearly equally important, but early usage is relatively more important. In addition, timely feedback may be important if a user behaves in a way that is detrimental to weight loss for a week. To address this, our model was developed to cover 3 types of sequence data (window, week, variables) to forecast changes from any week in the mobile program. Therefore, our model can provide timely feedback by predicting weight change from week to week.

Most of the variable-level attention weightings shown were either 1 or –1 (Figure 5). Each attention weighting needs to be understood to infer why the variable-level attention is represented as –1 or 1. The time-level attention weighting was always positive as it is derived from the softmax function, and the variable-level attention weighting ranged from –1 to 1 as it is derived from the hyperbolic tangent function, which is multiplied with the parameter *w* in linear transformation; therefore, we inferred that variable-level attention represented the sign of weighting at each time step in the model. Instead, parameter *w* in linear transformation seemed to represent the magnitude of weightings, and its values were constant regardless of the time step. Therefore, to at least understand both the magnitude and sign of the weightings (eg, regardless of the time step), variable-level attention weighting and *W* should be multiplied together.

Weight and meal inputs core behavior components of self-monitoring showed results consistent with those of previous studies [31-33]. A previous study [34] that did not consider timeseries identified factors contributing to weight loss using multivariate and univariate regression consistent with our findings; overall weight, meal input, and exercise input had a significant correlation with weight loss [34]. Similarly, calories were also a contributing factor to weight gain [34]. In our study, the weight, meal, and exercise inputs had consistent contribution coefficients of –0.021, –0.023, and –0.016, respectively, while caloric intake had a contribution coefficient of 0.100; these were found using interpretable RNNs, though our model cannot provide the statistical significance of contribution coefficient.

Furthermore, the yo-yo weight loss trajectory had a negative contribution coefficient of –0.006 for 8 weeks, then changed to a positive value of 0.06 at 8 weeks. To determine whether the weight loss trajectory is classified as a yo-yo or decreased pattern, the eighth week may be a critical week. In other words, to avoid a yo-yo trajectory, self-monitoring using the mobile app may be important especially for the initial 8 weeks. Meanwhile, because this model uses the historical shape of weight loss, there may be a concern that the model is weak because it indirectly learns the weight trajectory shape. Thus, we also conducted an experiment where the model without weight loss trajectories was retrained, and the result showed that the model maintained the robustness of performance (Multimedia Appendix 3). From the user’s weight entry, we derived and leveraged 2 variables—weight loss trajectory and weight input. In the real world, although coaches encourage users to enter their weight, there may be users without weight records. For users without any weight records, the accuracy and interpretation of the model’s prediction are limited. From the comparison between the model with and without weight loss trajectory as the feature (Multimedia Appendix 3), the trajectories may not be a high-contributing factor for predicting weight loss because the model compensates. Despite this, adding weight loss trajectory may provide a hint for early intervention with cluster assignment. For example, it is possible to coach the user by considering the user’s weight loss trajectory compared to the weight loss plateau or rebounding shape. Therefore, the selection of feature needs to be considered with the interpretability of each feature in mind.

Unexpectedly, drinking frequency and overall caloric intake did not make a consistent contribution to the model. This may be due to low retention, causing the number of drinks and overconsumption of calories not to be recorded because the records related to meal logs were not entered consistently within the 16 weeks.

When AI is used as a decision aid (eg, for a coach), an explanation can improve its trustworthiness [35]. For example, a coach (or another health provider) may highlight user’s behaviors that cause weight gain based on the interpretation of the model. Based on the feedback of intervention theory, feedback focused on the behavior task itself introduces the importance of timing in feedback delivery [36,37]. Also, a feedback strategy based on such a theory should be personalized and goal oriented. From this point of view, users may modify their behavior to increase weight loss based on the results of a real-time prediction and contribution factors, which improves their motivation [36,37].

### Limitations

Our study has some inherent limitations due to its implementation of interpretable AI using lifelog data. The individuals included in this study had purchased a subscription to a weight loss program and were selected if they had weight records for 16 weeks. This may result in selection bias wherein the chosen individuals were those with high adherence or the intention to lose weight. However, given that most of the participants who used other weight loss apps were in their forties, and a high rate of users were female in previous studies, characteristics similar to those in our study, our study is able to show robust findings with respect to population group that uses these apps [20,38-42]. Furthermore, given that the average 30-day retention rate is 3.4% for health and fitness apps in the United States, and that some researchers have reported a 3% to 8% 30-day retention rate for mental health apps, this app has an inherently higher retention rate (completion of 16-week program) compared to peer health care apps [43-45].

Despite this, there is limited research on predictive models for weight change using lifelog data and identification of contributing factors to weight loss. These results can make up for the lack of evidence on the prediction of weight change in an electronically delivered intervention setting and contribute to better outcomes. Furthermore, the implementation of the model was based on a single mobile app for obesity management. Therefore, for more generalized evidence, research using lifelogs from various apps is needed.

### Conclusion

In conclusion, we have demonstrated that an interpretable RNN can consider app usage behavior and the users’ demographic characteristics while maintaining model transparency.

## Appendix

Multimedia Appendix 1 Illustrated explanation of conditional RNN and explainability.

Multimedia Appendix 2 Clustered weight loss trajectories using k-means with dynamic time warping.

Multimedia Appendix 3 Model performance from 5 cross-validations after retraining the model without trajectory features.

## Abbreviations

AI: artificial intelligence
MAPE: mean absolute percentage error
RNN: recurrent neural network
RETAIN: reverse time attention model
