# Supplementary material for: Interpretable Conditional Recurrent Neural Network for Weight Change Prediction: Algorithm Development and Validation Study
Source: JMIR Mhealth Uhealth. 2021 Mar 29;9(3):e22183. doi: 10.2196/22183 (PMC8088842; doi:10.2196/22183)
Supplement: Multimedia Appendix 1 [file mhealth_v9i3e22183_app1.docx]

**Development and Validation of an Interpretable Conditional RNN for Weight Change Prediction Using an Obesity Management Mobile App**

Ho Heon Kim^1^RN MS; Young In Kim^1,2^MD; Yu Rang Park^1,*^PhD

**Illustrated explanation of conditional RNN and explainability**

1. Assume we have *N* time series related to application usage with *i* length, where $x_{i, N}^{(T)}=[x_{1,N},..x_{i,N}]$ and $x_{i, N}^{(T)}\in\mathbb{R}^{i}$, *k* time-fixed variables where $x^{(F)}\in\mathbb{R}^{k}$, and a target series y of length *i*, where $\mathbf{y}=[y_{1}, \ldots,y_{i}]$, and $\mathbf{y}\in\mathbb{R}^{i}$. By stacking *N* time series, we define a multi-variate input series $X_{i}=\{x_{i,1}^{\left( T \right)},\ldots,x_{i,N}^{\left( T \right)}\}$ and contemporary multi-variate inputs is denoted at time *i* by $x_{i}^{\left( T \right)}$.


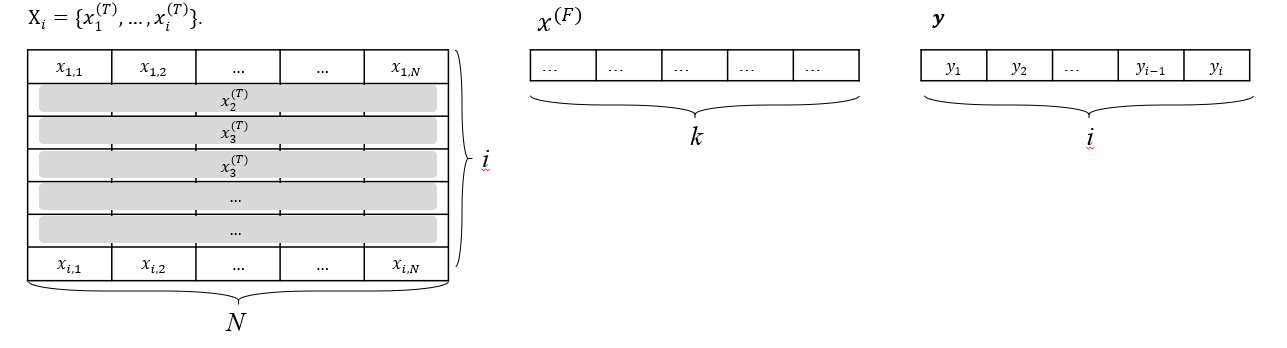


1. Given $X_{i}$ and $x^{(F)}$, we leverage two types of data for our model to predict contemporary target values using deep learning $\mathcal{F}$, namely $\hat{y}_{i}=\mathcal{F(}X_{i}$, $x^{(F)}$). The concatenation operation is denoted by $\oplus$ and element-wise multiplication is denoted by $⨀$. $A_{:i}$ is denote slice 1-D tensor A from first index to *i-*th index.
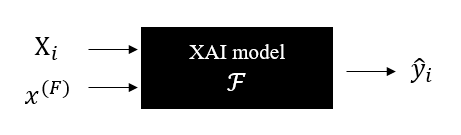

2. We attempt to predict the weight after a 16-week intervention using the output vector $y_{i}\in\{0, \infty\}$ by concatenating $c_{i}$ and $x^{(F)}$ and applying a linear transformation with *w* weight vector*,* where $w\in\mathbb{R}^{N+k}$. This is expressed as follows:

|  | $\hat{y}_{i}=w^{⊺}\left( c_{i}\oplus x^{(F)} \right)+b$ | (1) |
| --- | --- | --- |

where $c_{i}$ denotes the context vector $c_{i}\in\mathbb{R}^{N}$. $c_{i}$ is the sum of the time-level variables $x_{1,1}^{(T)},\ldots,x_{i,N}^{(T)}$ weighted by attention $\alpha_{i}$, $\beta_{i}$. Time-level attention weight $\alpha_{i}$ is a scalar value that reflects the relative importance of the data at time *I,* and it ranges from 0 to 1 (the sum of $\alpha_{i}$ is 1). $\beta_{i}$ is a vector that explains the importance of each value of $X_{i}$ within *i* time.

Time-level attention, and variable-level attention weights can be calculated from continuous variable without embedding layer in RETAIN such as app usage time series equation (2). Consistent with RETAIN, interpretation of the weight prediction model involves the time-level attention weight ($\alpha_{i}$) and variable-level attention weight ($\beta_{i}$) from each RNN $g_{i}, h_{i}$ where $\alpha_{i}$ is a scalar, and $\beta_{i}$ is a vector with N length. This suggests the importance of the input data at that time, compared to overall time steps. For example, “Weight gain can lead to chronic disease” can be classified as a negative sentence in sentiment analysis, and the relative importance of each word in this sentence can be obtained. “Chronic disease” is a word of high importance. The time-level attention weight was relatively important among all the time steps.

|  | $g_{i},g_{i-1},\ldots,g_{1}=\mathrm{RN}N_{\alpha}\left( X_{i},X_{i-1},\ldots X_{1} \right)$  $e_{j}=w_{\alpha}^{⊺}g_{j}+b_{\alpha}$ for *j* = 1,…,i  $\alpha_{1},\alpha_{2},\ldots,\alpha_{i}=\mathrm{Softmax}\left( e_{1},e_{2},\ldots,e_{i} \right)$  $h_{i},h_{i-1},\ldots,h_{1}=\mathrm{RN}N_{\beta}\left( X_{i},X_{i-1},\ldots X_{1} \right)$  $\beta_{j}=\tanh\left( W_{\beta}h_{j}+b_{\beta} \right)$ | (2) |
| --- | --- | --- |

1. Below is a visualized matrix operation above the equation. We get each contribution coefficient of the time-fixed variable $w_{N+1}^{⊺}$. As each *m-*th time-fixed variable multiplied by the *N+m* th value in matrix $w^{⊺}$, the $w_{N+1:}^{⊺}$ can play the role of or act as a coefficient in linear regression (beige colored box).
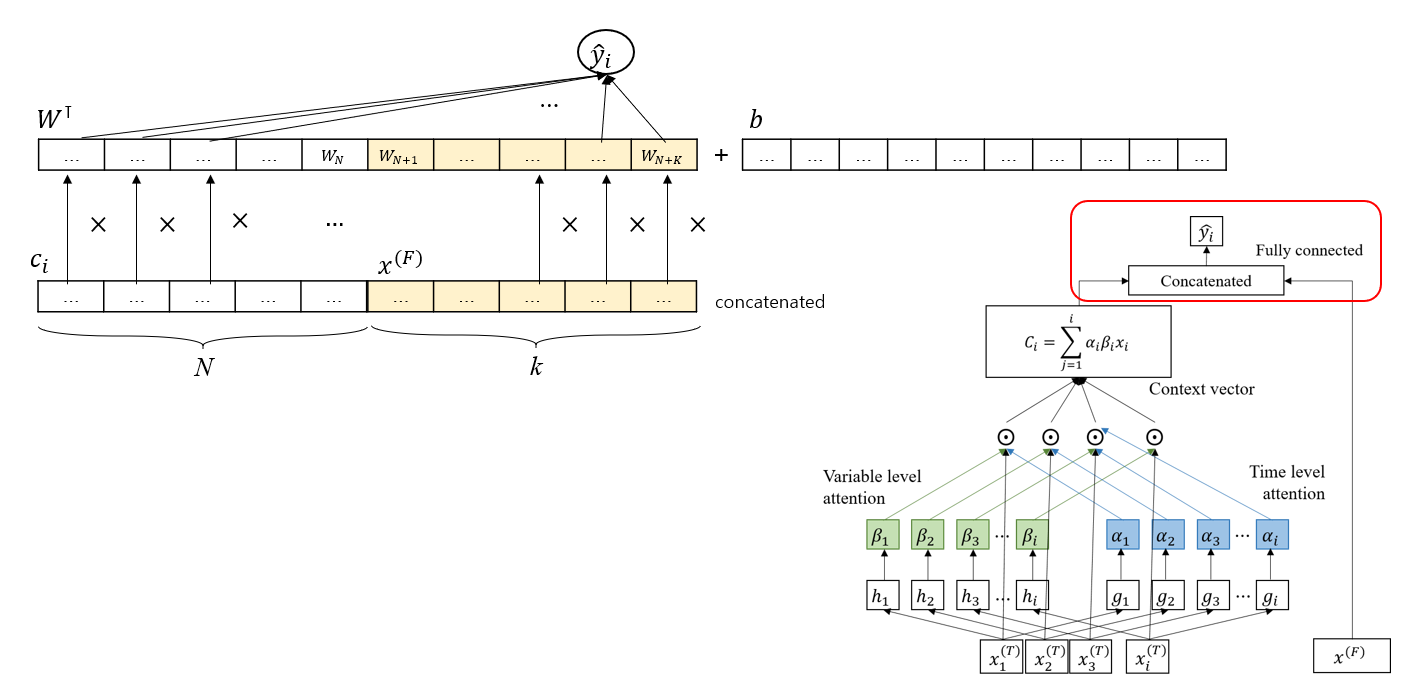

2. Further, equation (1) can be rewritten, and the contributions of the predicted value of the model can be calculated as equation (3). In addition, the operation-related time-variant variables can be illustrated as shown in the following figure. For each time i, the time level attention weight ($\alpha_{i})$ can be calculated, and its ranked values ranged from 0 to 1 (their sum is 1) as illustrated by the beige, green, blue, and yellow boxes.

| $\hat{y}_{i}= w^{⊺}\left( \sum_{j=1}^{i} \alpha_{j}\beta_{j}{⨀x}_{j}^{\left( T \right)}\oplus x^{\left( F \right)} \right)+b$  $=w_{:N}^{⊺}\left( \sum_{j=1}^{i} \alpha_{j}\beta_{j}{⨀x}_{j}^{\left( T \right)} \right)+ {w_{N:}^{⊺}x}^{\left( F \right)}+b$  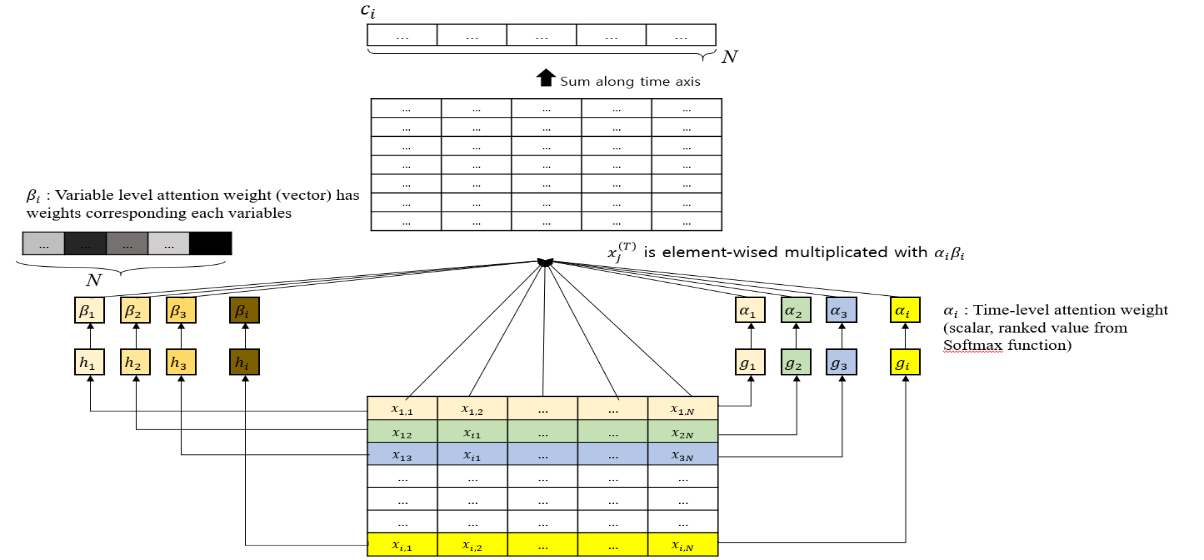 | (3) |
| --- | --- |

For each time j, variable level attention weight ($\beta_{j})$can be calculated (vector). Each element $\beta_{j,N}$ in the vector corresponds to each element of the time-variant variable at j time. Context $c_{i}$ can be derived element-wise by multiplying each time-variant variable$x_{j}^{\left( T \right)}$ with $\alpha_{j}\beta_{j}$ and sum along the time axis. Reversely, we can decompose context $c_{i}$ and get the coefficient value corresponding to each element of time variable x multiplied from the following equation (4).

|  | $\omega\left( \hat{y}_{i}, x_{j,N} \right)^{(T)}= w_{N}^{⊺}\alpha_{j}\beta_{j,N}x_{j,N}$ | (4) |
| --- | --- | --- |
