# Supplementary material for: Interpretable Conditional Recurrent Neural Network for Weight Change Prediction: Algorithm Development and Validation Study
Source: JMIR Mhealth Uhealth. 2021 Mar 29;9(3):e22183. doi: 10.2196/22183 (PMC8088842; doi:10.2196/22183)
Supplement: Multimedia Appendix 2 [file mhealth_v9i3e22183_app2.docx]

Multimedia appendix 2. Clustered weight loss trajectories using K-means with DTW


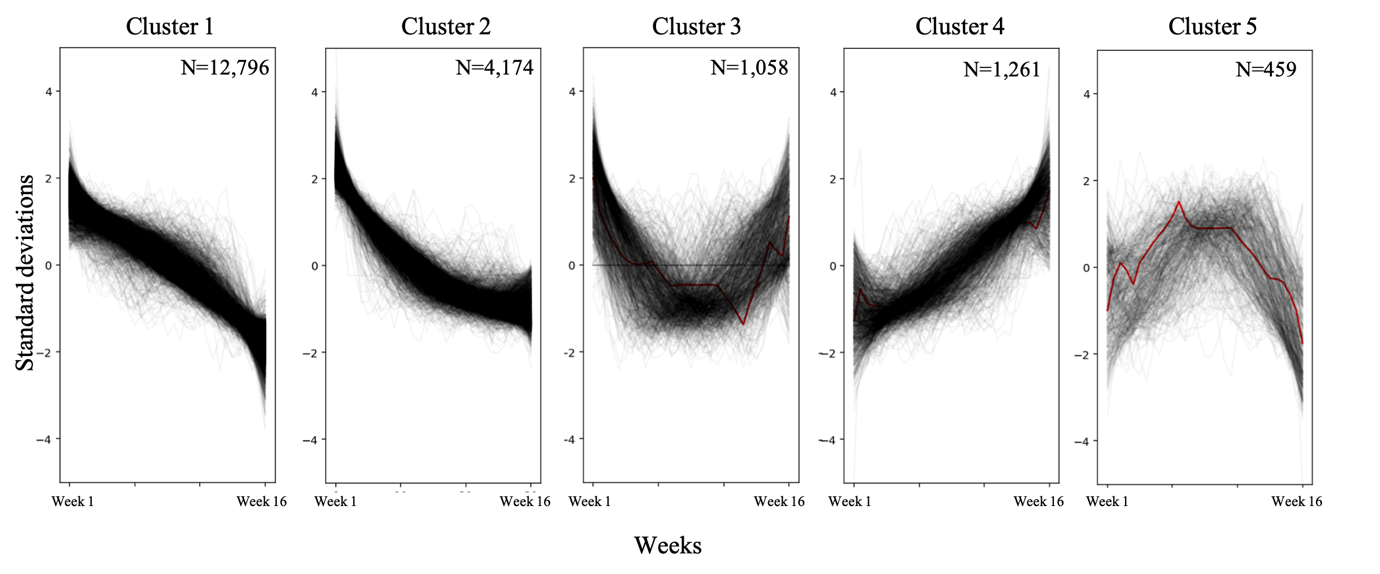


* Each black line signifies an individual user’s weight loss journey. The red line represents weight loss trajectories of each cluster.
