# Supplementary material for: Interpretable Conditional Recurrent Neural Network for Weight Change Prediction: Algorithm Development and Validation Study
Source: JMIR Mhealth Uhealth. 2021 Mar 29;9(3):e22183. doi: 10.2196/22183 (PMC8088842; doi:10.2196/22183)

Multimedia appendix 3

**Figure A-1.** Model performance (MAPE) from 5 cross validations after retraining the model without trajectory features.


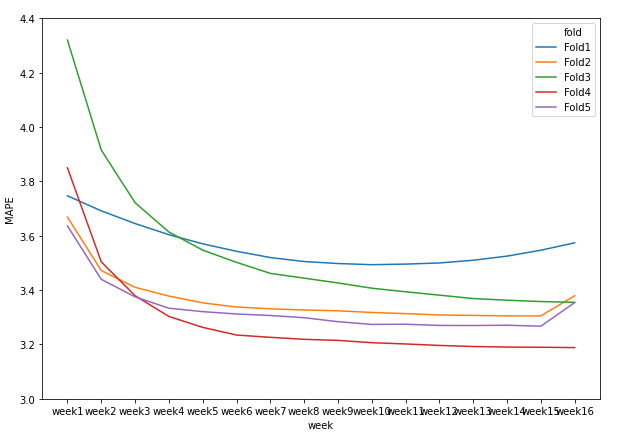

Supplement: Multimedia Appendix 3 [file mhealth_v9i3e22183_app3.docx]
